# An epidemiological comparative study on diagnosis of rodent leptospirosis in Mazandaran Province, northern Iran

**DOI:** 10.4178/epih/e2015012

**Published:** 2015-02-23

**Authors:** Behzad Esfandiari, Mohammad Reza Pourshafie, Mohammad Mehdi Gouya, Pejvak Khaki, Ehsan Mostafavi, Jamshid Darvish, Soheila Moradi Bidhendi, Hamed Hanifi, Hossein Nahrevanian

**Affiliations:** 1 Department of Epidemiology, Pasteur Institute of Iran, Tehran, Iran; 2 Department of Bacteriology, Pasteur Institute of Iran, Tehran, Iran; 3 Centre for Diseases Control and Prevention, Ministry of Health, Tehran, Iran; 4 Microbiology Department, Razi Vaccine and Serum Research Institute, Karaj, Iran; 5 Department of Biology, Ferdowsi University of Mashhad, Mashhad, Iran; 6 Department of Parasitology, Pasteur Institute of Iran, Tehran, Iran

**Keywords:** Culture, Iran, *Leptospira*, Microscopic agglutination test, Mazandaran, Nested polymerase chain reaction, Rodent

## Abstract

**OBJECTIVES:**

Leptospirosis is a zoonosis caused by leptospires, in which transmission occurs through contact with contaminated biological fluids from infected animals. Rodents can act as a source of infection for humans and animals. The disease has a global distribution, mainly in humid, tropical and sub-tropical regions. The aim of this study was to compare culture assays, the microscopic agglutination test (MAT), polymerase chain reaction (PCR), and nested PCR (n-PCR), for the diagnosis of leptospirosis in rodents in Mazandaran Province, northern Iran.

**METHODS:**

One hundred fifty-one rodents were trapped alive at 10 locations, and their urine and kidney samples were collected and used for the isolation of live *Leptospira*. The infecting serovars were identified and the antibody titres were measured by MAT, using a panel of 20 strains of live *Leptospira* species as antigens. The presence of leptospiral DNA was evaluated in urine and kidney samples using PCR and n-PCR.

**RESULTS:**

No live leptospires were isolated from the kidney and urine samples of the rodents. Different detection rates of leptospirosis were observed with MAT (21.2%), PCR (11.3%), and n-PCR (3.3%). The dominant strain was *Leptospira serjoehardjo* (34.4%, p=0.28), although other serotypes were also found. The prevalence of positive leptospirosis tests in rodents was 15.9, 2.6, and 2.6% among *Rattus norvegicus, R. rattus*, and *Apodemus sylvaticus*, respectively.

**CONCLUSIONS:**

Leptospirosis was prevalent in rodents in Mazandaran Province, northern Iran. MAT was able to detect leptospires more frequently than culture or PCR. The kidney was a more suitable site for identifying leptospiral DNA by n-PCR than urine. Culture was not found to be an appropriate technique for clinical diagnosis.

## INTRODUCTION

Leptospirosis (also known as rice field fever) is one of the most important common zoonotic diseases. Since it can be transmitted through a wide range of hosts, it has a wide distribution worldwide. The infectious agent can be transmitted directly or indirectly from livestock to humans, and causes two clinical phases of disease, known as the icteric and anicteric forms of the disease. Most animals affected by leptospirosis remain carriers throughout their lifetime, periodically excreting bacteria in their urine. Most pathogenic leptospires can remain alive in water and soil for months and can enter into humans or other animal hosts through scratches in the skin [1,2].

In Iran, the temperate and humid climate zone ranges from the plains along the Caspian Sea to the northern foothills of the Alborz mountain range. In this region, rice planting is the predominant occupation of the rural population, and the majority of farmers keep one or more livestock animals in their houses, such as cows, sheep, dogs, or horses. In most villages in this region, stagnant pools of water or rivers and pounds are used for irrigation. In this region, the overall environment is suitable for leptospirosis to spread into humans [3].

Following the description of leptospirosis by Johnson [4], serobacteriological studies have been conducted in most countries. These studies have found a high prevalence of leptospirosis in many countries in a range of domestic and wild mammals [1]. Ever since the first reported case of leptospirosis in Iran was described in 1956, several other reports from various regions of Iran have been published [5]. In these studies, several thousand sera samples from cattle, sheep, and camels were analysed using the microscopic agglutination test (MAT), showing that 31% of cows and 17% of sheep were infected with *Leptospira grippotyphosa, L. pomona*, or *L. icterohaemorrhagiae* [6]. Since 1997, the disease has been reported in agricultural workers in the city of Rasht in Gilan Province every year during the cultivation season. A surge of suspected leptospirosis cases relative to previous years was reported in June and July 1998 [3].

The diagnosis of leptospirosis is on the basis of serological findings from plasma, cerebrospinal fluid, and urine cultures. Most diagnosis assays do not isolate leptospires because of the cost, the complexity of the media, and the incubation period. Therefore, serology is an important method in the diagnosis of leptospiral infection. Currently, the most reliable diagnostic method is based on the detection of specific serum antibodies. MAT is the gold-standard method recommended by the World Health Organization. Although the advantage of MAT is its specificity, combined with a high sensitivity for serovars, it is complex and costly, which limits its global implementation [7,8]. Alternative molecular diagnostic methods, such as polymerase chain reaction (PCR) and real-time PCR, have been developed to detect leptospires in the first stage of infection. In some cases, leptospiral DNA has been detected in the blood and serum of patients in the early stage of infection, when MAT was not able to diagnose the disease [9].

The majority of the population of Mazandaran Province in northern Iran work in rice cultivation and/or animal husbandry, which provide a perfect niche for the growth and spread of leptospires in animals and humans [3]. In order to reduce the prevalence of leptospirosis, infected animals must be properly identified, using a combination of culture, serology, and PCR tests. The purpose of this study was to conduct a comparative study on culture assays, MAT, and PCR as methods of diagnosing rodent leptospirosis in Mazandaran Province, northern Iran.

## MATERIALS AND METHODS

### Study area and sample collection

**Figure 1.**
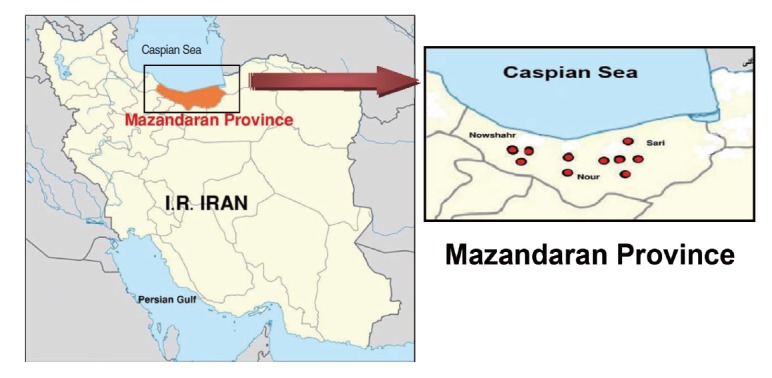
Map of the study area and sampling locations in Mazandaran Province, northern Iran.

This descriptive cross-sectional study was carried out during the summer of 2013 in Mazandaran Province in northern Iran. In this study, active colonies of rodents were trapped in 10 geographical areas around three major cities, resulting in a total of 151 rodents (Figure 1). The rodents were categorized according to characteristics such as gender, genus, species, and geographical origin. The animal experiments were performed according to ethical guidelines designed to protect the animals from further pain or discomfort. The study was approved by the institutional ethics review board of the Pasteur Institute of Iran, where the work was performed.

### Preparation of blood samples

Animals were terminally anaesthetized and ethically sacrificed. Anaesthesia was carried out by the inhalation of diethyl ether (Sigma-Aldrich, St. Louis, MO, USA) and 5 mL of blood was taken into a syringe by cardiac puncture. The blood samples were transferred into two tubes. One sample was treated with heparin to extract the DNA for PCR assays, and the other sample was placed into a tube without heparin, in order to separate the serum for MAT. The non-heparin samples were centrifuged at 3,000 g for 10 minutes at room temperature. The sera samples were kept in sterile 1.5 mL micro-tubes at -20°C until used. Death occurred after the blood samples were taken from the rodents.

### Urine sample collection

The single animal method involves allowing a single mouse or rat to urinate on a plastic cling container outside of the animal cage. When they were picked up by the experimenter, they passed drops of urine and were collected with an automatic pipette; otherwise, in some cases, it was possible to collect urine directly from the bladder using a syringe during kidney collection.

### Kidney tissue preparation and microbial culture assay

The kidneys of all rodents were removed after terminal anaesthesia. Bacterial culture was performed using the Ellinghausen-McCullough-Johnson-Harris (EMJH) method [10,11]. Briefly, samples were immediately diluted in a 1:1 ratio of 0.01 M phosphate-buffered saline with a pH of 7.4 and centrifuged at 3,500 g for 10 minutes. The pellet was double-inoculated in EMJH medium supplemented with 5-fluorouracil and phosphomycin disodium. Pellet samples were observed by dark field microscopy at 40× to search for leptospires. Cultures were incubated at 28°C and evaluated weekly to search for turbidity or the formation of a Dinger growth ring. Cultures with these appearance were assessed by taking a small sample and observed by dark field microscopy for the confirmation of leptospires. *Leptospira* isolates were preserved in glycerol (25% by volume) at -70°C. Iranian Type Culture Collection strains were used as positive controls. *Escherichia coli* were used as negative controls [10,11].

### Microscopic agglutination test

MAT was performed on the sera samples collected, using 20 live leptospiral strains as antigens. The strains belonged to the following serogroups: *Australis* (strain Jez Bratislava), *Autumnalis* (Akiyami A), *Ballum* (Mus 127), *Bataviae* (Swart), *Canicola* (Hond Utrecht IV), *Icterohaemorrhagiae* (RGA), *Grippotyphosa* (Moskova V), *Hebdomadis* (Hebdomadis), *Javanica* (Poi), *Pomona* (Pomona), *Pyrogenes* (Pyrogenes), and *Semaranga* (Patoc I). MAT was applied at successively doubled dilutions, starting from 1:20. Positive samples were titrated to their end point. All the strains were maintained in EMJH medium with periodical subculture. Cultures seven days old without contamination were utilized for MAT [10,11].

### Polymerase chain reaction (PCR) and nested PCR

DNA from the collected kidney tissues and urine samples was purified using the Qiagen extraction kit (Qiagen Strasse, Hilden, Germany), after which they were dissolved in Tris-EDTA buffer and kept at -20°C. The DNA was quantified by agarose gel electrophoresis and spectrophotometric analysis was performed by calculating the A 260/A 280 ratios and the A 260 values to determine protein impurities and DNA concentrations. In order to investigate the presence of different species of *Leptospira*, a kit was used that allowed both PCR and nested PCR (n-PCR) to be performed (Accupower PCR preMix, Bioneer, Seoul, Korea). For amplification of DNA, a primer set based on the *Lipl32* target gene was employed. These primers amplified all pathogenic and non-pathogenic *Leptospira* species. For this purpose, 15 μL of distilled water, 75 μL of each primer solution (25 μM), and 1 μL of DNA were added into micro-tubes. The temperature profile was as follows: one cycle at 94°C for 3 minutes, 35 cycles at 94°C for 30 seconds each, 30 seconds at 52°C, 1.5 minutes at 72°C, and a final extension at 72°C for 7 minutes. The *Lipl32* gene was amplified by a first cycle at 94°C for 5 minutes, followed by 35 cycles at 94°C for 90 seconds, 90 seconds at 51°C, 2 minutes at 72°C, and a final extension at 72°C for 7 minutes. The sequences of the forward and reverse PCR primers were 5´CCTAACTAAGGAGAGTCTATG-3´ and 5´-TTACTTAGTCGCGTCAGAAGC-3´, respectively. For the n-PCR assay, the primers were 5´-CCTAACTAAGGAGAGTCTATG-3´ and 5´-GAATCAAGATCCCAATCCTC-3´ as the forward primers and 5´-TTACTTAGTCGCGTCAGAAGC-3´ and 5´-AGATCCGTAGGGAAGTAACG-3´ as the reverse primers (designed by the Leptospira Reference Laboratory, Razi Vaccine and Serum Research). The PCR results were determined using electrophoresis in 1% agarose gel [11].

### Data analysis

Statistical analysis was performed in two stages, using SPSS version 17 (SPSS Inc., Chicago, IL, USA). The first stage was a descriptive analysis that aimed to characterize the study sample. The goal of the second stage was to correlate all statistical variables and parameters.

## RESULTS

### Epidemiological parameters

In Mazandaran Province, MAT found that 21.2% of all rodent samples tested positive for *Leptospira* serotypes, of which 11.9% were in the Nowshahr district, 6.6% were in the Nour district, and 2.6% were in the Sari district. The most common *Leptospira* serotype was determined to be *L. serjoehardjo* (34.4%, p=0.28) (Table 1). The findings of this study showed that there was no statistically significant difference in the distribution of different *Leptospira* serotypes between rural and urban areas. The percentage of rodents with positive test results was 3.3 and 17.9% in urban and rural areas, respectively, which was found to be a statistically significant difference (Table 2). No statistically significant association was found between leptospirosis prevalence and the gender of the rodents. Of the rodents identified, 76.8% belonged to the species *Rattus norvegicus*, 6.0% belonged to the species *R. rattus*, and 17.2% belonged to the species *Apodemus sylvaticus*. Positive results for leptospirosis were found in 15.9% of the R. norvegicus sample, 2.6% of the *R. rattus* sample, and 2.6% of the *A. sylvaticus* sample (Table 3). The detection rate of *Leptospira* serotypes in sera samples varied according to the use of different assays: MAT returned positive results in 21.2% of rodents, PCR returned positive results in 11.3% of rodents, and 3.3% of rodents tested positive using n-PCR. These results are consistent with previous studies [12,13] (Table 4). MAT anti-*Leptospira* antibody titre results showed that 79.0% of rodents did not test positive for any antibody titre, while 21.2% were positive at antibody titres ≥ 1:200. Broken down by species, antibody titre results showed that 15.9% of the *R. norvegicus* sample, 2.6% of the *R. rattus* sample, and 2.6% of the *A. sylvaticus* sample tested positive. The distribution of positive results according to anti-*Leptospira* antibody titres was as follows: 10.6% tested positive at 1:200, 6.6% tested positive at 1:400, 3.3% tested positive at 1:800, and 0.7% tested positive at 1:1,600 (Table 5).

### Culture assay

Cultures were considered negative and discarded after eight weeks of culture with no growth. No leptospires were observed in any urine and kidney samples using dark field microscopy. Difficulties in isolating leptospires may be due to several factors, including the low number of micro-organisms, the short period of excretion in urine, the loss of bacteria in continuous culture, inappropriate techniques for hunting the animals, and sampling time.

### Serological assay

Positive MAT assays at dilutions ranging from 1:200 to 1:1,600 were considered to indicate a positive test for leptospirosis. Among the 151 samples, 32 (21.2%) were positive and 119 (78.8%) were negative. The species detected in this study were *L. autumnalis* (3.3%), *L. canicola* (0.7%), *L. grippotyphosa* (1.3%), *L. serjoehardjo* (7.3%), *L. icterohaemorrhagiaecopenhagen* (3.3%), *L. baiium* (0.7%), *L. australis* (2.0%), *L. lai* (0.7%), and *L. cynopteri* (2%).

### Polymerase chain reaction (PCR) and nested PCR

**Figure 2.**
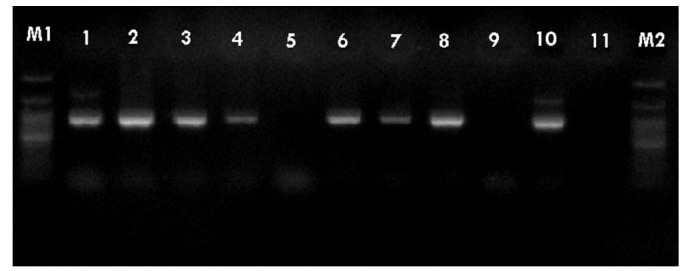
Sample of polymerase chain reaction results using the F1R1 primer (1,021 base pairs). Columns M1 and M2, DNA ladder (100 base pairs); columns 1-4 and 6-8, positive samples; columns 5 and 9, negative samples; column 10, positive control; column 11, negative control.

**Figure 3.**
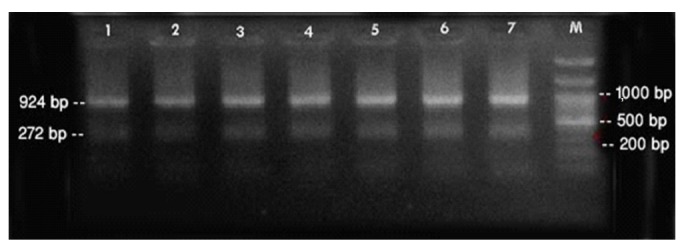
Sample of nested-polymerase chain reaction products using the F1R1 and F2R2 primers. Column M, DNA ladder (100 bp); columns 1-7, positive samples. bp, base pairs.

PCR was performed using the primer pairs described above. A 1,021 base pair represented the *Lipl32* band, which is a feature of *Leptospira*. It was found that 10.6% of the kidney samples (16 out of 151) showed positive results using PCR. Only one urine sample (0.7%) returned a positive result. The n-PCR assay, using two primers (F1R2 and F1R1), resulted in five samples (3.3%) testing positive. Altogether, in this study, PCR and n-PCR were able to detect leptospirosis in 11.3% and 3.3% of rodents in Mazandaran Province, respectively (Figures 2 and 3).

## DISCUSSION

Leptospirosis is a widespread zoonotic disease, which is common in humid tropical, semitropical, and temperate climates, with wild and domestic animals and rodents as sources of disease [1]. The plains along the Caspian Sea in northern Iran have a temperate climate and humid conditions, which are appropriate for *Leptospira* infection. In this region, the predominant occupation is rice cultivation, and most farmers keep livestock, including cows, horses, and dogs, in their houses. These conditions are suitable for the prevalence of human leptospirosis [3].

In this study, MAT detected leptospires in 21.2% of rodents in Mazandaran Province, with the highest positive rates observed in the Nowshahr district, followed by the Nour and Sari districts. The most common *Leptospira* serotype in these areas was determined to be *L. serjoehardjo*. As well, a statistically significant (p<0.05) difference in the prevalence of leptospires was found between urban and rural areas, which is probably due to the lack of hygiene and the presence of both domestic and wild animals as disease reservoirs in rural areas. No statistically significant association was found between the prevalence of leptospirosis and the gender of the rodents (p>0.05). The highest rates of infection were identified among *R. norvegicus*, with lower rates observed among *R. rattus* and *A. sylvaticus*.

MAT is considered the gold-standard reference method for diagnosing leptospirosis, and is based on the binding of *Leptospira*-specific antibodies to antigens, which is more sensitive than PCR and n-PCR. The results of this study further confirm this assessment.

In most occupational studies amongst leptospirosis patients, farmers have been shown to have the highest incidence of leptospirosis. Bharti et al. [1] stated that the most important risk factors for this disease are occupational factors, direct or indirect contact with animals or their dead bodies, contact with grass and bushes, swimming, hunting, aquatic sports, and traveling to hot and rainy areas; these findings show that occupational factors play an important role in the spread of leptospirosis. The first comprehensive study on leptospirosis in Iran was conducted in 1957, as reported by Rafyi & Maghami [6] in the Razi Vaccine and Serum Research Institute in Iran evaluated 3,000 sera samples from cattle, sheep, and camels using MAT. Their results indicated that 31% of cows and 17% of sheep were infected with *L. grippotyphosa, L. pomona* or *L. Icterohaemorrhagiae*.

Several molecular typing methods have been employed to test for the presence of leptospires. Terpstra et al. [13] were the first to use the dot-blot hybridization method, with probes marked with P32 and biotin, for the detection of leptospires. Millar et al. [14] used the same method for tracking and identifying leptospires directly from clinical specimens. DNA hybridization has also been used to determine genetic relatedness amongst *Leptospira* species. Ramadass et al. [15] proposed removing the prefix *hardjo* from the strain name *hardjobovis* and calling it *L. borgpetersoni* serovar *hardjo* strain *bovis*, because they did not find any direct genetic relation between the *L. hardjobovis* and *L. hardjoprajitno* strains. In the molecular typing method, the complete genome of leptospires is cut by one or more restriction endonuclease enzymes, after which the banding patterns are used for the recognition of serovars in restriction endonuclease analysis. Restriction endonuclease analysis was used by Marshall et al. [16] to separate the *L. icterohaemorrhagiae* and *L. hebdomadis* serovars. Other researchers, such as Thiermann et al. [17], have used restriction endonuclease analysis to classify the leptospiral isolates belonging to serogroup *Pomona*, and Ellis et al. [18,19], were able to serogroup 300 species of *Leptospira*. Comprehensive restriction endonuclease analysis patterns for *Leptospira* have been generated by using 20 endonuclease enzymes.

In recent years, PCR-based methods have become more popular, cheaper, and easier. Although MAT is still the gold standard for the serological diagnosis of leptospirosis, the interpretation of MAT results is also very useful in other applications, especially in the differentiation of cross-reactivity amongst different serogroups in complicated cases, particularly in clinical samples taken during the acute stage of the disease [20]. This study demonstrated that simple PCR may be more sensitive than n-PCR, as the n-PCR and culture techniques had many more false negative results than MAT.

However, this study had some limitations that should be considered before drawing any broad conclusions, including single-sampling and the fact that we ignored the possible role of other animals as reservoirs due to financial considerations. The negative results of the *Leptospira* cultures may have occurred due to the use of inappropriate techniques to hunt the animals, the sampling time, the short period of excretion in urine, the low number of micro-organisms, and the loss of bacteria in continuous culture. Our results suggest that detecting and analysing pathogenic leptospiral serovars in temperate plains environments, such as that found in Mazandaran Province, is an important health issue. The seroprevalence of leptospirosis in Mazandaran Province is increasing, and more research must be conducted in order to clarify the epidemiological picture of leptospirosis in Iran.

## Figures and Tables

**Table 1.** The proportions of *Leptospira* serotypes among the total samples identified in the major locations of Mazandaran Province

Serotype	Sampling location			Total
	Sari	Nour	Nowshahr	
Negative	64 (94.1)	20 (66.7)	35 (66.0)	119/151 (78.8)
Positive	4 (5.9)	10 (33.3)	18 (34.0)	32/151 (21.2)
*L. autumnalis*	1 (0.7)	1 (0.7)	3 (2.0)	5 (3.31)
*L. canicola*	0 (0.0)	1 (0.7)	0 (0.0)	1 (0.7)
*L. grippotyphosa*	0 (0.0)	2 (1.3)	0 (0.0)	2 (1.3)
*L. serjoehardjo*	2(1.3)	3 (2.0)	6 (4.0)	11 (7.3)
*L. icterohaemorrhagiaecopenhagen*	1 (0.7)	1 (0.7)	3 (2.0)	5 (3.3)
*L. icterohaemorrhagia icterohaemorrhagiae*	0 (0.0)	0 (0.0)	0 (0.0)	0 (0.0)
*L. baiium*	0 (0.0)	0 (0.0)	1 (0.7)	1 (0.7)
*L. australis*	0 (0.0)	2 (1.3)	1 (0.7)	3 (2.0)
*L. pyrogenes*	0 (0.0)	0 (0.0)	0 (0.0)	0 (0.0)
*L. sejroesejroe*	0 (0.0)	0 (0.0)	0 (0.0)	0 (0.0)
*L. javanica*	0 (0.0)	0 (0.0)	0 (0.0)	0 (0.0)
*L. bataviae*	0 (0.0)	0 (0.0)	0 (0.0)	0 (0.0)
*L. sejroewolfi*	0 (0.0)	0 (0.0)	0 (0.0)	0 (0.0)
*L. tarrasovie*	0 (0.0)	0 (0.0)	0 (0.0)	0 (0.0)
*L. lai*	0 (0.0)	0 (0.0)	1 (0.7)	1 (0.7)
*L. cynopteri*	0 (0.0)	0 (0.0)	3 (2.0)	3 (2.0)
*L. pomona*	0 (0.0)	0 (0.0)	0 (0.0)	0 (0.0)
*L. hepdomatis*	0 (0.0)	0 (0.0)	0 (0.0)	0 (0.0)
*L. panama*	0 (0.0)	0 (0.0)	0 (0.0)	0 (0.0)
*L. djasimin*	0 (0.0)	0 (0.0)	0 (0.0)	0 (0.0)
Total	68/151 (45.0)	30/151 (19.9)	53/151 (35.1)	151 (100.0)

Values are presented as number (%).

**Table 2.** The proportions of *Leptospira* serotypes among the total samples obtained in urban and rural areas

Serotype	Sampling location		Total
	Urban	Rural	
Negative	52 (91.2)	67 (71.3)	119/151 (78.8)
Positive	5 (8.8)	27 (28.7)	32/151 (21.2)
*L. autumnalis*	0 (0.0)	5 (3.3)	5 (3.3)
*L. canicola*	0 (0.0)	1 (0.7)	1 (0.7)
*L. grippotyphosa*	0 (0.0)	2(1.3)	2 (1.3)
*L. serjoehardjo*	3 (2.0)	8 (5.3)	11 (7.3)
*L. icterohaemorrhagiae copenhagen*	1 (0.7)	4 (2.7)	5 (3.3)
*L. icterohaemorrhagia icterohaemorrhagiae*	0 (0.0)	0 (0.0)	0 (0.0)
*L. baiium*	0 (0.0)	1 (0.7)	1 (0.7)
*L. australis*	0 (0.0)	3 (2.0)	3 (2.0)
*L. pyrogenes*	0 (0.0)	0 (0.0)	0 (0.0)
*L. sejroesejroe*	0 (0.0)	0 (0.0)	0 (0.0)
*L. javanica*	0 (0.0)	0 (0.0)	0 (0.0)
*L. bataviae*	0 (0.0)	0 (0.0)	0 (0.0)
*L. sejroewolfi*	0 (0.0)	0 (0.0)	0 (0.0)
*L. tarrasovie*	0 (0.0)	0 (0.0)	0 (0.0)
*L. lai*	0 (0.0)	1 (0.7)	1 (0.7)
*L. cynopteri*	1 (0.7)	2 (1.3)	3 (2.0)
*L. pomona*	0 (0.0)	0 (0.0)	0 (0.0)
*L. hepdomatis*	0 (0.0)	0 (0.0)	0 (0.0)
*L. panama*	0 (0.0)	0 (0.0)	0 (0.0)
*L. djasimin*	0 (0.0)	0 (0.0)	0 (0.0)
Total	57/151 (37.7)	94/151 (62.3)	151 (100.0)

Values are presented as number (%).

**Table 3.** The proportions of *Leptospira* serotypes among the total samples according to the host species

Serotype	Host species			Total
	*Rattus norvegicus*	*Rattus rattus*	*Apodemus sylvaticus*	
Negative	92 (79.3)	5 (55.6)	22 (84.6)	119/151 (78.8)
Positive	24 (20.7)	4 (44.4)	4 (15.4)	32/151 (21.2)
*L. autumnalis*	5 (3.3)	0 (0.0)	0 (0.0)	5 (3.3)
*L. canicola*	0 (0.0)	0 (0.0)	1 (0.7)	1 (0.7)
*L. grippotyphosa*	1 (0.7)	0 (0.0)	1 (0.7)	2 (1.3)
*L. serjoehardjo*	9 (6.0)	1 (0.7)	1 (0.7)	11 (7.3)
*L. icterohaemorrhagiae copenhagen*	2 (1.3)	2 (1.3)	1 (0.7)	5 (3.3)
*L. icterohaemorrhagia icterohaemorrhagiae*	0 (0.0)	0 (0.0)	0 (0.0)	0 (0.0)
*L. baiium*	1 (0.7)	0 (0.0)	0 (0.0)	1 (0.7)
*L. australis*	3 (2.0)	0 (0.0)	0 (0.0)	3 (2.0)
*L. pyrogenes*	0 (0.0)	0 (0.0)	0 (0.0)	0 (0.0)
*L. sejroesejroe*	0 (0.0)	0 (0.0)	0 (0.0)	0 (0.0)
*L. javanica*	0 (0.0)	0 (0.0)	0 (0.0)	0 (0.0)
*L. bataviae*	0 (0.0)	0 (0.0)	0 (0.0)	0 (0.0)
*L. sejroewolfi*	0 (0.0)	0 (0.0)	0 (0.0)	0 (0.0)
*L. tarrasovie*	0 (0.0)	0 (0.0)	0 (0.0)	0 (0.0)
*L. lai*	0 (0.0)	1 (0.7)	0 (0.0)	1 (0.7)
*L. cynopteri*	3 (2.0)	0 (0.0)	0 (0.0)	3 (2.0)
*L. pomona*	0 (0.0)	0 (0.0)	0 (0.0)	0 (0.0)
*L. hepdomatis*	0 (0.0)	0 (0.0)	0 (0.0)	0 (0.0)
*L. panama*	0 (0.0)	0 (0.0)	0 (0.0)	0 (0.0)
*L. djasimin*	0 (0.0)	0 (0.0)	0 (0.0)	0 (0.0)
Total	116/151 (76.8)	9/151 (6.0)	26/151 (17.2)	151 (100.0)

Values are presented as number (%).

**Table 4.** Comparison of the detection of *Leptospira* serotypes by MAT, PCR, and nested PCR in sera samples

Serotype	Assay			Total
	MAT	PCR	Nested PCR	
*L. autumnalis*	5 (3.3)	2 (1.3)	0 (0.0)	7 (4.6)
*L. canicola*	1 (0.7)	0 (0.0)	0 (0.0)	1 (0.7)
*L. grippotyphosa*	2 (1.3)	1 (0.7)	0 (0.0)	3 (2.0)
*L. serjoehardjo*	11 (0.0)	3 (2.0)	1 (0.7)	15 (9.9)
*L. icterohaemorrhagiae copenhagen*	5 (3.3)	3 (2.0)	1 (0.7)	9 (6.0)
*L. icterohaemorrhagia icterohaemorrhagiae*	0 (0.0)	0 (0.0)	0 (0.0)	0 (0.0)
*L. baiium*	1 (0.7)	1 (0.7)	1 (0.0)	3 (2.0)
*L. australis*	3 (2.0)	3 (2.0)	0 (0.0)	6 (4.0)
*L. pyrogenes*	0 (0.0)	0 (0.0)	0 (0.0)	0 (0.0)
*L. sejroesejroe*	0 (0.0)	0 (0.0)	0 (0.0)	0 (0.0)
*L. javanica*	0 (0.0)	0 (0.0)	0 (0.0)	0 (0.0)
*L. bataviae*	0 (0.0)	0 (0.0)	0 (0.0)	0 (0.0)
*L. sejroewolfi*	0 (0.0)	0 (0.0)	0 (0.0)	0 (0.0)
*L. tarrasovie*	0 (0.0)	0 (0.0)	0 (0.0)	0 (0.0)
*L. tarrasovie*	0 (0.0)	0 (0.0)	0 (0.0)	0 (0.0)
*L. lai*	1 (0.7)	1 (0.7)	0 (0.0)	2 (1.3)
*L. cynopteri*	3 (2.0)	3 (2.0)	2 (1.3)	8 (5.3)
*L. pomona*	0 (0.0)	0 (0.0)	0 (0.0)	0 (0.0)
*L. hepdomatis*	0 (0.0)	0 (0.0)	0 (0.0)	0 (0.0)
*L. panama*	0 (0.0)	0 (0.0)	0 (0.0)	0 (0.0)
*L. djasimin*	0 (0.0)	0 (0.0)	0 (0.0)	0 (0.0)
Total detected	32/151 (21.2)	17/151 (11.3)	5/151 (3.3)	54/151 (35.8)

Values are presented as number (%).

MAT, microscopic agglutination test; PCR, polymerase chain reaction.

**Table 5.** Associations between microscopic agglutination test anti-*Leptospira* antibody titers and rodent species

	*Rattus norvegicus *(n = 116)	*Rattus rattus* (n = 9)	*Apodemus sylvaticus *(n = 26)	Total (n = 151)
1:1,600	1 (0.9)	0 (0.0)	0 (0.0)	1 (0.7)
1:800	4 (3.5)	0 (0.0)	1 (3.9)	5 (3.3)
1:400	8 (6.9)	1 (11.1)	1 (3.9)	10 (6.6)
1:200	11 (9.5)	3 (33.1)	2 (7.7)	16 (10.6)
Positive	24 (20.7)	4 (44.4)	4 (15.4)	32 (21.2)
Negative	92 (79.3)	5 (55.6)	22 (84.6)	119 (78.8)

Values are presented as number (%).
